# Nature’s Timepiece—Molecular Coordination of Metabolism and Its Impact on Aging

**DOI:** 10.3390/ijms14023026

**Published:** 2013-01-31

**Authors:** Andrea Bednářová, Dalibor Kodrík, Natraj Krishnan

**Affiliations:** 1 Institute of Entomology, Biology Centre, Academy of Science, Branišovská 31, České Budějovice 370 05-CZ, Czech Republic; E-Mails: bednarovaandrea@centrum.cz (A.B.); kodrik@entu.cas.cz (D.K.); 2 Faculty of Science, South Bohemian University, Branišovská 31, České Budějovice 370 05-CZ, Czech Republic; 3 Department of Biochemistry, Molecular Biology, Entomology and Plant Pathology, Mississippi State University, Mississippi State, MS 39762, USA

**Keywords:** aging, circadian clocks, cellular metabolism, homeostasis, metabolic hormones, oxidative stress, reactive oxygen species

## Abstract

Circadian rhythms are found in almost all organisms from cyanobacteria to humans, where most behavioral and physiological processes occur over a period of approximately 24 h in tandem with the day/night cycles. In general, these rhythmic processes are under regulation of circadian clocks. The role of circadian clocks in regulating metabolism and consequently cellular and metabolic homeostasis is an intensively investigated area of research. However, the links between circadian clocks and aging are correlative and only recently being investigated. A physiological decline in most processes is associated with advancing age, and occurs at the onset of maturity and in some instances is the result of accumulation of cellular damage beyond a critical level. A fully functional circadian clock would be vital to timing events in general metabolism, thus contributing to metabolic health and to ensure an increased “health-span” during the process of aging. Here, we present recent evidence of links between clocks, cellular metabolism, aging and oxidative stress (one of the causative factors of aging). In the light of these data, we arrive at conceptual generalizations of this relationship across the spectrum of model organisms from fruit flies to mammals.

## 1. Introduction

Circadian rhythms are observed in a wide variety of biological, physiological and metabolic processes in most organisms. These rhythmic cycles are governed by an endogenous circadian clock. Thus, a crucial function of the endogenous circadian system would be to anticipate and adapt to environmental changes in light, temperature, food and even mate availability, and organize behavior and physiology to these changing situations. In addition, the circadian system plays a regulatory role by the temporal coordination of physiological, cellular and molecular processes such that synergistic processes are timed to coincide, whereas processes that are conflicting are temporally separated. There is increasing evidence that a smoothly running endogenous clock is crucial for energy balance in organisms from cyanobacteria to mammals [1–4]. Diurnal rhythms are entrained by exogenous Zeitgebers (time-givers), especially light/dark (LD) cycles. However, many rhythms also persist in constant darkness (DD) with a periodicity of circa 24 h, due to their endogenous nature. The rhythm can also be entrained by changes in timing of food and temperature (although the period of rhythm can be stable over a wide range of temperature—*i.e.*, it can be temperature compensated) [5]. The importance of the circadian clock for organismal homeostasis was demonstrated by loss of sleep/wake cycles and metabolic defects following targeted disruption of core clock genes in mice [6,7]. Additional roles of clocks in basic cellular pathways have also been elucidated such as regulation of nutrient and energy balance, cell cycle, DNA-damage repair and xenobiotic detoxification [6,8,9]. In view of these data, it should not come as a surprise that disruption of circadian rhythms would increase vulnerability to stressors, accelerate aging and could lead to various pathologies including cancer [10–13].

## 2. The Molecular Clock Network and Homeostasis

Circadian rhythms have been described in both prokaryotes and eukaryotes, and many molecular features associated with the circadian clock are evolutionarily conserved [14]. The molecular clock network consists of transcriptional and translational feedback loops where core clock genes are transcribed and these translational products inhibit their own transcription. These timed transcriptional and translational feedback processes generate the molecular rhythms, which eventually translate to the circadian rhythms observed in most organisms. The basic organization of the circadian clock has a hierarchy—with every cell in the organism having an autonomous clock, and different cells and tissues are synchronized to oscillate with the same phase. This orchestration occurs via the “central” or “master” clock, which is entrained by input from zeitgebers. Genetic analyses have revealed numerous clock genes in different species and most studies to date have focused on understanding the molecular mechanisms of the rhythms generated by these core clock genes. In the fruit fly *Drosophila melanogaster*, by far the best-studied organism with regard to molecular circadian functioning, the clock system is highly similar to the vertebrate circadian clock [15]. A network of ~150 pacemaker neurons in the brain regulates rest/activity rhythms, which comprise the central clock [16]. The peripheral clocks are located in many cells of the nervous system, retinal photoreceptors, sensory neurons, Malpighian tubules, gut, fat body, *etc.* [17–19]. The core molecular circadian cellular clock consists of CLOCK/CYCLE heterodimer at the node of all interacting feed-back loops. The main players are PERIOD, TIMELESS, VRILLE, PDP1ɛ and CLOCKWORK ORANGE molecules in addition to highly important kinases and phosphatases, that is, DOUBLETIME, CASEIN KINASE 2, SHAGGY, PROTEIN PHOSPHATASE 1 and PROTEIN PHOSPHATASE 2A. The sub-cellular localization and activities of clock proteins are controlled by post-translational modifications, particularly phosphorylation, mediated by specific kinases and phosphatases [20]. Synchronization of the clocks phase to external light/dark (LD) cycle is mediated by a blue light photoreceptive flavin-binding protein CRYPTOCHROME (CRY) [21]. For progression of the circadian cycle, the clock proteins are targeted for degradation by the Ubiquitin-proteasome system [22]. In *Caenorhabditis elegans*, many of the homologs of the *Drosophila* clock proteins are involved in development and developmental timing. A recent whole-transcriptome study evaluating circadian variations in transcript levels reported that all canonical clock gene homologs in *C. elegans* are noncycling [23]. Despite these results, there is evidence that *lin-42*, the *period* homolog in *C. elegans* plays a role in nematode circadian rhythms [24]. Similarly *Tim-1* corresponds to *timeless*, *Aha-1* corresponds to *CLOCK*, *CYCLE* and *CLOCKWORK ORANGE*. The *C. elegans* CES-2 is a basic leucine zipper (bZIP) protein that is the best homolog of *D. melanogaster* PDP1ɛ, similarly *Atf-2* is the homolog of *vrille* [25]. Of the *D. melanogaster* kinases and phosphatases playing a role in the circadian central oscillator, *doubletime* corresponds to both *C. elegans kin-20* and *kin-19*, *shaggy* is similar to *gsk-3*, casein kinase 2 consists of *kin-3* and *kin-10*, protein phosphatase 1 has two corresponding genes *gsp-1* and *gsp-2* and the protein phosphatase regulatory subunit is encoded by *sur-6* [26,27].

In mammals, the circadian clock resides in the hypothalamic suprachiasmatic nucleus (SCN), which is recognized as being the master clock. The clock network also exists in almost all peripheral tissues, including liver, heart, kidney and blood cells [28–31]. Although the SCN is not essential for driving peripheral oscillations, it appears to coordinate peripheral clocks [31]. At the molecular level, the circadian clock consists of an auto-regulatory feed-back loop regulated by specific proteins that are rhythmically translated in an integrated manner [32]. These transcriptional—translational auto-regulatory feedback loops have both positive and negative elements [33,34]. The positive components are two basic helix-loop-helix (bHLH) transcription factors called CLOCK and brain and muscle Arnt-like protein 1 (BMAL1, homologous to CYCLE in *Drosophila*) [35,36]. The heterodimer activates the transcription of several other clock genes including *Period* (*PER*) and *Cryptochrome* (*CRY*) [37–39]. The resultant PER and CRY proteins form a heterodimer, translocate to the nucleus, and inhibit the activity of CLOCK-BMAL1, thus forming a negative feedback loop. The intracellular clock is thought to directly and/or indirectly regulate the expression of numerous genes [33,34].

The control of circadian rhythms is driven by the transcriptional-translational feedback loops that ensure homeostasis; any dysfunctions in such rhythms have been implicated in serious pathologies such as sleep disorders, cardiovascular diseases, depression, cancer, response to oxidative stress and accelerated aging [40–45]. The complexity underlying the mechanism that drives circadian rhythms is one of the key challenges on drawing direct connections between the detailed pathway of how the biologic clock contributes to events that modulate both cellular and metabolic homeostasis. Despite this there is a growing body of evidence that circadian rhythms are connected with the physiological state of an organism, e.g., its nutritional condition, hormonal fluctuation, aging and also certain pathologies. These physiological states might act positively or negatively on the biologic clocks, but these events must be integrated and fine-tuned to ensure homeostasis. The circadian clock must be tightly regulated to modulate a state of dynamic homeostasis at the cellular and metabolic level, which implies that there is a physiological integration of metabolic and circadian rhythms all directed to the maintenance of a fully functioning organism and any disruption of this coordinated control would impact aging.

## 3. The Integration of Metabolic and Circadian Rhythms for Homeostasis

A central feature in energy homeostasis is the temporal coordination of metabolism. Circadian clocks in fruit flies help coordinate rhythmic feeding behavior and regulate energy consumption and metabolism. Food consumption in *Drosophila* occurs consistently at specific times of the day (primarily in the morning) and this rhythm in feeding behavior persists in constant darkness [46]. The fat body (a homolog of the mammalian liver and adipose tissue) of flies harbor clocks which are involved in regulating fuel storage and energy balance; the antennae, maxillary palp and proboscis all harbor odor/gustatory receptors under control of clock which regulate taste and feeding, the gastrointestinal tract involved in digestion and nutrient absorption is also under regulation of clocks. Selective and targeted disruption of clock functions in any of these tissues results in disrupted feeding behavior. In case of the nematode *C. elegans* although circadian rhythms have been described earlier at the behavioral level, yet these rhythms appear to be relatively non-robust. In addition, while the *C. elegans* genome is predicted to encode homologs of most *Drosophila* and mammalian core clock components, the roles of these genes appear to be largely restricted to development [47]. Unlike *Drosophila* or mammals, temperature plays an important role in driving the expression of a large set of genes implicated in metabolic processes in *C. elegans* [23]. In mammals, diurnal oscillations of various nutrients, blood glucose, lipids and hormones are accompanied by rhythmic activation of diverse metabolic pathways. Transcriptome profiling studies have revealed that the fraction of cyclically expressed transcripts in each peripheral tissue ranges between 5% and 10% of the total population and the vast majority of these genes are tissue specific [48–56]. Many hormones involved in metabolism such as insulin [57], glucagon [58], adiponectin [59], corticosterone [60], leptin, and ghrelin [61], have been shown to exhibit circadian oscillation. These studies indicate that circadian signals play a crucial role in control of metabolic rhythms, which ensures energy and nutrient homeostasis at both cellular and organismal levels. The concentrations of various metabolites are sensed by a unique class of transcriptional regulators—the nuclear hormone receptors [62–64]. A complex interaction between circadian clock and nuclear receptor signaling exists, which enables rhythmic cycles of metabolites in key metabolic tissues. Nuclear receptors such as REV-ERBα and REV-ERBβ and RORα, β and γ are directly linked to BMAL1 and CLOCK *via* interconnecting positive and negative transcriptional feedback loops [65–67]. Moreover, nuclear receptors implicated in metabolic control such as PPARα and TRα have been shown to act as indirect mediators of BMAL1 and CLOCK to carry out specific outputs in a circadian manner [64,68]. In addition, PER2 itself has been shown to propagate clock information to metabolic genes by directly interacting with and acting as a co-regulator of nuclear receptor-mediated transcription [69]. The rhythmic expression and activity of the metabolic pathways is mainly attributed to the coordinated expression of clock genes along with rhythmic metabolite sensing by nuclear receptors which then feed back again on the clock. With metabolic rhythms and circadian clocks feeding back on each other, the output contributes to cellular homeostasis [70]. The prominent role of the circadian clock in energy metabolism is further demonstrated by obesity and metabolic disorders developed in some clock mutant or knockout mice.

## 4. Coordination of Central and Peripheral Clocks to Organize Metabolism during Aging

A number of experimental evidences have demonstrated a strong link between metabolism and circadian rhythms and that circadian clocks control the levels of many cellular and circulating metabolites. Despite this, the coupling between metabolism and the circadian clock is complex with more evidence accumulating in favor of a dynamic feed-back loop, wherein the rhythm impacts the metabolic activity and metabolism feeds back to impinge upon the rhythm [71]. While a connection between metabolism and peripheral clocks is more easily apparent, a connection between metabolism and the SCN is not as obvious, because the former are responsive to nutritional stimuli. Several lines of evidence point to a functional link between metabolism and circadian rhythms in the brain. The expression of certain genes encoding metabolic enzymes and transport systems for energy metabolites are under circadian control e.g., glycogen phosphorylase [72,73], cytochrome oxidase, lactate dehydrogenase, *etc.* The concentration of cellular ATP exhibits a marked fluctuation as a function of light/dark cycle in the SCN, and this in turn stimulates neural activity [74]. Thus, a relationship between neural activity rhythms and metabolic activity rhythms might provide the link between stimuli such as light, food, running, or other sensory stimuli and entrainment of circadian oscillators in the brain. The SCN resets, or entrains peripheral clocks by mechanisms that are not fully understood. While the core circadian clock is common to all tissues whether central or peripheral, the output clock (which governs clock controlled genes- CCGs), or cell processes regulated by the clock, may be tissue, or cell-type specific. Despite this, there is coordination between central and peripheral clocks to ensure a fine tuned, organized metabolism, which would profoundly impact the process of aging.

### 4.1. Aging, ROS and the Circadian Connection

**Figure 1:**
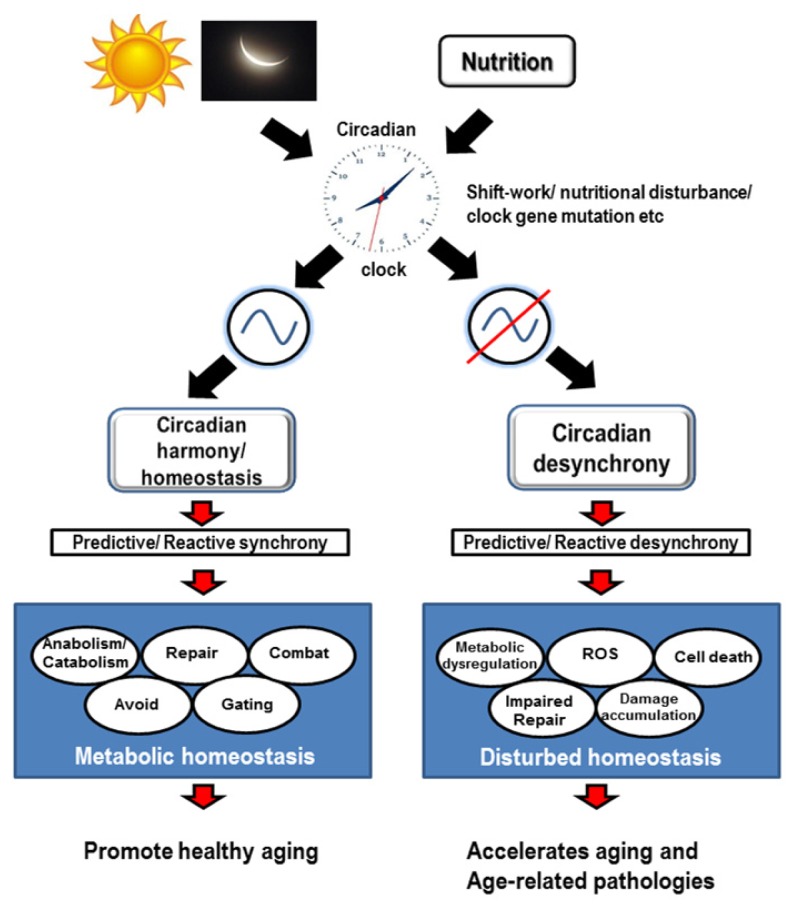
A schematic representation of the role of clocks in cellular tasks resulting from predictive/reactive synchrony or desynchrony impacting cellular homeostasis and consequently affecting healthy aging. Light-dark cycles as well as nutrition play an important role as zeitgebers (time-givers) resulting in synchronous functioning of the clock and clock controlled genes (CCGs) which regulate metabolism by coordinating anabolic and catabolic reactions, repair functions, combating/avoiding unfavorable stress situations as well as gating specific physiological, cellular and molecular events in a temporal fashion to achieve cellular metabolic homeostasis. Situations which impact the clock, such as shift-work, disturbance in the nutritional cues of even mutations in certain clock genes result in circadian desynchrony impairing the organisms ability to predict/react to changing demands of physiology which unbalances cellular homeostasis by metabolic dysregulation, reactive oxygen species (ROS) generation, affecting repair mechanisms leading to damage accumulation and apoptosis. This results in accelerated aging and propensity for age-related pathologies.

Aging is characterized by decrements in maximum function and accumulation of mitochondrial DNA mutations, which are best observed in organs such as the brain that contain post-mitotic cells. Oxygen radicals are increasingly considered responsible for part of these aging changes. Comparative studies of animals with different aging rates have shown that the rate of mitochondrial oxygen radical generation is directly related to the steady-state level of oxidative damage to mitochondria and inversely correlated with maximum longevity in higher vertebrates. These two traits link oxidative stress with aging. Many of the detrimental changes associated with aging such as decline in neurotransmitters, reduced motor and sensory systems, fragmented sleep, memory and learning, a gradual decline in general metabolism are linked to the generation of reactive oxygen species (ROS) by mitochondria [75]. In principle, oxidative stress could be related to aging through variations in ROS generation, ROS elimination, or both. Despite this, the rate of aging is not controlled by antioxidants alone, since studies in which genes encoding particular antioxidants are knocked out do not seem to have a major change in their rates of aging compared with ones with intact antioxidant genes [76–78]. Increased mean lifespan (MLS) is an index more frequently used than increased maximum lifespan. The increases in MLS suggest that antioxidants can non-specifically protect against many causes of early death—and increase survival—when experiments are performed under sub-optimal conditions. The connections between circadian rhythmicity and energy balance (metabolism) are particularly evident in aging studies. While neurodegenerative diseases and aging frequently coincide with disrupted circadian rhythms and fragmented sleep patterns [79], the most convincing evidences for linking aging phenotype to disrupted circadian rhythms comes from *Bmal1^−/−^* mice and *per^01^* fruit flies [12,44]. The mutant mice and fruit flies show distinct signs of early aging and have a reduced lifespan compared to the normal counterparts. The tissues of *Bmal1^−/−^* mice and brains of *per^01^* fruit flies also show a high accumulation of ROS, which is consistent with the idea that *Bmal1* or *per* (in case of fruit flies) participates in oxidative stress response [42,43]. *C. elegans* also has been reported to show a daily variation in response to oxidative and osmotic stress, which provides evidence of rhythmic underlying gene expression which governs these responses to stress [80]. Due to the involvement of mitochondria in oxidative phosphorylation and respiration, these powerhouses of the cell are subject to high levels of ROS production, an inevitable byproduct of respiration. As a result, mitochondrial proteins are central targets of oxidative damage and, when damaged, may contribute to aging disorders such as neurodegeneration [45]. This vicious cycle of ROS-induced damage to mitochondrial DNA, which inevitably leads to reduced respiration efficiency and a further increase in the level of intracellular ROS production is thought to be central to the aging process and the accumulating ROS in the brain probably lead to the age-related neurodegenerative pathologies of Alzheimer’s and Parkinson’s disease. The circadian clock is geared to anticipate any changes in all levels of biological organization and tends to maintain homeostasis. Hence, any state that tends to pull away from the state of homeostasis would be a “stress”, whether it is oxidative stress, ionic or nutritional imbalance or even abrupt and unpredictable changes in the environment. Thus, circadian clocks maintain temporal coordination of the internal milieu whereby conflicts with external or internal environment is lessened and a circadian harmony is created. Additionally circadian rhythms can enhance the efficiency of the component elements of the homeostatic defense that are enhanced when it is most likely or most regularly to be required. Hence, both “reactive” as well as “predictive” homeostatic mechanisms are regulated by the circadian clocks and this ensures a healthy life span during aging (Figure 1).

### 4.2. Cell Cycle Control and Circadian Clocks

The mammalian CLOCK and BMAL1 proteins have been implicated to play a role in cell cycle control. Embryonic fibroblasts isolated from *Clock* mutant mice demonstrate a delayed cell cycle entrance after serum starvation. The expression of many genes such as cell cycle inhibitors *p21* and *p27* was up-regulated, while *Cdk2* and *cyclins D3* and *E1* were downregulated in *Clock* mutant cells [81]. Thus, these mutations may also impact aging through their effects on cell-cycle regulation, checkpoint proteins and DNA damage repair all of which would have an impact on genome integrity and the aging phenotype.

## 5. Linking Regulators of Aging and the Circadian Clock

The concept that circadian clocks located in the SCN could actually regulate the process of aging is not new [82,83], yet tangible evidences on such a control mechanism has not been forthcoming till recently. Now, there has been certain exciting development in the field of aging and circadian rhythm researches, which brings up important insights into the fascinating connection between physiological rhythmicity, metabolism and aging. These recently investigated factors that regulate aging have links to circadian clocks, and have been briefly dealt with below:

### 5.1. The NAD^+^ and Sirtuins Connection

The mitochondrion is the seat of cellular respiration, energy generation and is also crucial for cellular metabolism. Carrier molecules such as ATP and NAD^+^ are used in oxidation reduction reactions that are critical for maintenance of energy balance. NAD^+^ is derived from niacin and operates as a coenzyme for multiple cellular dehydrogenases, and its subsequent reduction to NADH precedes its subsequent oxidation by the respiratory chain. Hence, the role of NAD^+^ as a hydrogen carrier is vital for the production and maintenance of energy stores. Importantly, nicotinamide mononucleotide adenylyl transferase-1 (Nmnat1), an enzyme involved in the biosynthesis of NAD^+^, protects against axonal degeneration in Wallerian degeneration slow mice (Wld^s^). These mice have a spontaneous mutation that increases activation of the Nmnat1 protein resulting in elevated NAD^+^ levels and consequent silent information regulator1 (Sirt1)- dependent protection against axonal injury [84,85]. The observation that Sirt1 activity oscillates suggests that there may be a link between neuroprotection, redox state and circadian rhythmicity [86]. This link is also supported by observations in *Drosophila*, where administration of paraquat results in reduced clock gene cycling in peripheral tissues and a forkhead box O protein (FOXO)- dependent sensitivity to oxidative stress in the central pacemaker [87]. These studies strongly indicate the importance of the circadian clock in the control of metabolism and aging but also beg the question of what is the level of their interaction at the cellular level.

**Figure 2:**
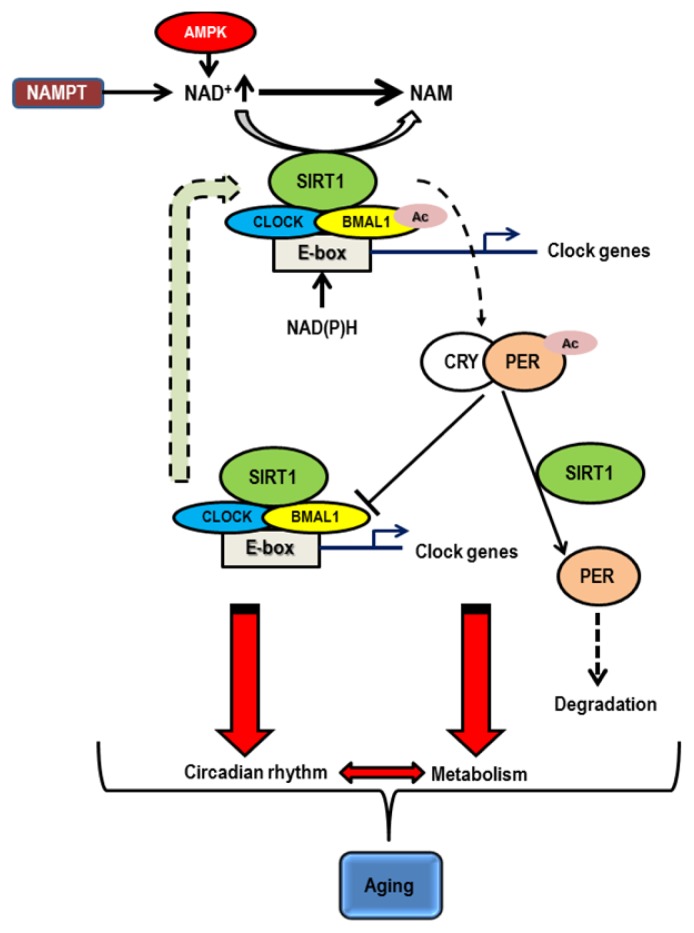
A representation of SIRT1- mediated regulation of the circadian clock and metabolism impacting aging. SIRT1 interacts with CLOCK, which heterodimerizes with BMAL1. BMAL1 is deacetylated by SIRT1 in a circadian manner and promotes expression of clock genes. Additionally, SIRT1 interacts with and deacetylates PER2 (in mammals) which heterodimerizes with CRY proteins to inhibit CLOCK-BMAL1 function and promotes its degradation. NAD^+^ from AMPK and NAMPT is a key regulator for SIRT1 function. Synchronous expression of clock genes results in regulation of clock controlled genes (CCGs) which impact metabolism and hence aging.

An important regulator that has recently attracted major attention in the field of aging research is the SIR2 (Silent Information Regulator 2) protein family, now known as “sirtuins” [88–92]. These are an evolutionarily conserved family of NAD-dependent protein deacetylases/ADP-ribosyltransferases. In *Saccharomyces cerevisiae*, *C. elegans* and *D. melanogaster*, SIR2 and its orthologs regulate aging and longevity [93–97]. There are seven mammalian sirtuins, SIRT1 through SIRT7, and the majority of mammalian sirtuin research has so far focused on the function of the SIR2 ortholog SIRT1 [89,90,92]. While it has not been demonstrated that SIRT1 regulates aging and longevity in mammals, it has been established that SIRT1 plays an important role in the regulation of metabolism in response to nutritional availability in multiple tissues. SIRT1 coordinates physiological programs that allow animals to survive through nutritionally scarce conditions by mediating critical metabolic responses to nutritional cues, particularly in low nutritional conditions such as fasting and caloric restriction [98,99]. This unique aspect of SIRT1 function and the absolute requirement of NAD for its activity place this protein at a central position as a key regulator that connects metabolism and aging. Nakahata *et al.* [86] found that SIRT1 shows the oscillation of its enzymatic activity and deacetylates both histone H3 and BMAL1, one of the critical regulators of the core clock mechanism, in a circadian manner. SIRT1 physically interacts with CLOCK, another key clock regulator that heterodimerizes with and acetylates BMAL1, and is recruited to the CLOCK:BMAL1 complex as the promoters of circadian clock genes. Asher *et al.* [100] showed that SIRT1 interacts with and deacetylates PER2, resulting in its degradation. Thus, SIRT1 regulates the amplitude and the duration of circadian gene expression through deacetylation of key circadian clock regulators, such as BMAL1 and PER2 (Figure 2). These studies have demonstrated the first connection between key regulators of aging and circadian rhythm. Furthermore, the core molecular clock machinery has also been demonstrated to be one of the most powerful modifiers of metabolism [6,101].

### 5.2. PGC-1α

**Figure 3:**
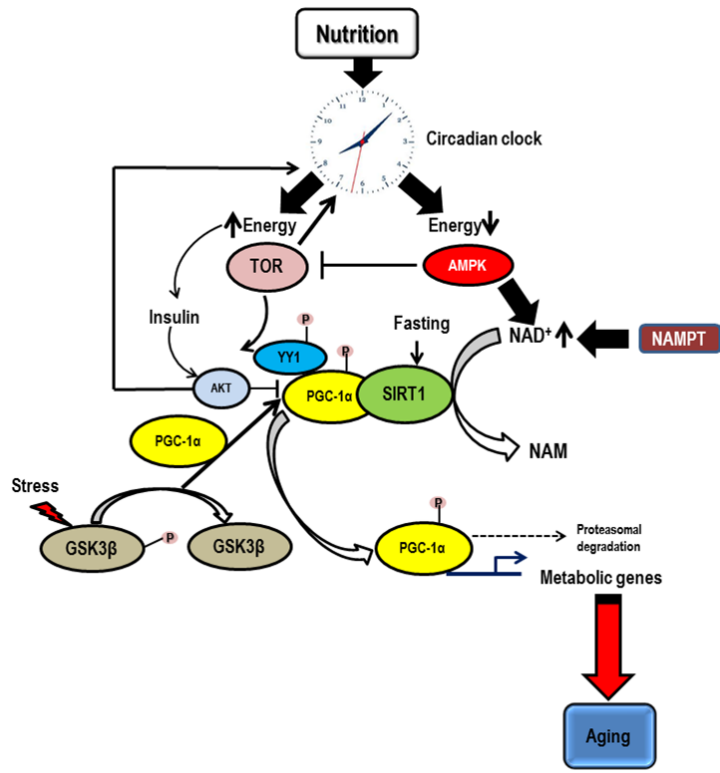
Regulation of PGC-1α—this schematic represents the relationship between nutrition, circadian clock and other regulatory factors involved in post-translational modification of PGC-1α and its consequent impact on metabolic genes and aging. Nutritional availability impacts the circadian clock - when energy is replete, TOR results in transcriptional regulation of PGC-1α through transcription factor YY1 (see text for details). The serine threonine kinase AKT (a key component of insulin signaling pathway) has an inhibitory effect (negative regulator) on PGC-1α. In energy deficient situations, AMPK has an inhibitory effect on TOR thus inducing PGC-1α and increased mitochondrial biogenesis. SIRT1 has a stimulatory deacetylation effect on PGC-1α under conditions of fasting and oxidative stress and helps accumulate it in the nucleus. Both TOR and AKT feed-back on the circadian clock and regulate it as a pacemaker. The nutrient sensitive kinase GSK3β targets PGC-1α for proteasomal mediated degradation and is a function of oxidative stress response. PGC-1α by its transcriptional activity plays an important role in the expression and activity of many metabolic genes which impact longevity.

Liver specific deletion of *Bmal1* causes loss of rhythmic expression of clock-regulated metabolic genes in the liver and hypoglycemia in the fasting phase of the daily feeding cycle [2]. Hepatic *Bmal1* expression is also regulated by PGC-1α (Peroxisome Proliferator Activated Receptor Gamma coactivator 1 Alpha), another SIRT1 target transcription factor that regulates glucose production in the liver and liver-specific PGC-1α knockdown in mice proves that this factor is required for clock function [102]. The transcriptional co-activator PGC-1α is a critical regulator of mitochondrial energy metabolism and biogenesis. Three members of this family have been identified based on sequence similarity to the founding member PGC-1α. PGC-1β is larger than PGC-1α but shares functionally equivalent protein domain features and has many common downstream targets [103]. Both PGC-1α and PGC-1β are expressed preferentially in high oxidative capacity tissues [104]. PRC-1 (PGC-1 related coactivator) is larger again with similar protein domain features but is not enriched in skeletal muscle and heart [105]. Although functionally similar, PGC-1α, PGC-1β and PRC-1 play distinct tissue specific roles in response to stimuli. At this point of time, PGC-1α is the best characterized and can be linked to aging and also as a transcriptional co-activator of nuclear receptors involved in multiple aspects of metabolism, PGC-1α may play a pivotal role in organismal metabolic homeostasis [106]. Remarkably, PGC-1α is responsive to multiple diverse stimuli: (i) changes in nutrient availability [107], (ii) oxidative species [108–110], (iii) endocrine signaling through insulin [111], (iv) circadian clock [102], and (v) energetic demand [112] among others. The ability of PGC-1α to respond to these disparate signals indicates that it is the principle medium through which external stimuli are communicated to the mitochondria. Some of the key regulatory factors for PGC-1α activity are as described below (Figure 3).

#### 5.2.1. AMPK

AMP-activated protein kinase (AMPK) is involved in adaptive response to energy deficit. Activation of AMPK induces PGC-1α. AMPK is involved in PGC-1α autoregulation; direct phosphorylation by AMPK promotes PGC-1α dependent induction at the PGC-1α promoter [113]. In addition to activating PGC-1α, AMPK also inhibits mTOR in mammals [114], a nutrient activated factor that regulates PGC-1α gene expression [115]. This dual arrangement allows for a PGC-1α response to energy deficit and to increased nutrient availability. AMPK acts to restore energy balance by enhancing oxidative metabolism, lipid utilization, promoting mitochondrial biogenesis and by inhibiting energy consuming processes. An important mechanism of AMPK function is increasing levels of NAD^+^, which in turn promote SIRT1 activity (Figure 3). This is accomplished by a pathway promoting mitochondrial fatty acid oxidation [116].

AMPK is also closely tied to clock function, particularly via its interactions with SIRT1 [116] and also through its phosphorylation and destabilization of CRY proteins [117], which involves ubiquitination of CRY1 by the F-box and leucine rich repeat protein, FBXL3. Nuclear localization of AMPK and CRY proteins showed inverse circadian phase [118]. Activation of AMPK caused phase advance in cell culture clock genes in mice [6]. Mice with disrupted AMPKγ3 subunit express impaired clock induction of muscle genes and circadian shifts in energy metabolism [119]. Importantly, these findings also suggest that cryptochromes may be a significant and previously unappreciated mediator of AMPK-dependent metabolic regulation with impact on gluconeogenesis.

#### 5.2.2. TOR

Target of rapamycin (TOR) is a nutrient sensing kinase that promotes cell growth in conditions of nutrient abundance [120]. Inhibition of TOR extends lifespan in yeast, worms and flies [121]. In mammalian cells, PGC-1α gene transcription is regulated by mTOR through interaction of PGC-1α with transcription factor YY1 and mTOR complex I [115]. The ability of cells to respond to changes in nutritional status through TOR is highly conserved and the role of this nutrient sensitive kinase in regulating mitochondrial function is likely to be important in the aging process [122].

#### 5.2.3. SIRT1

Activity of PGC-1α is regulated through inhibitory acetylation by GCN5 [123] and stimulatory deacetylation of SIRT1 [124–126]. PGC-1α is regulated by cellular localization, and in response to oxidative stress accumulates in the nucleus in a SIRT1 dependent manner [108]. Deacetylation of PGC-1α is required to sequester it in the nucleus and this cellular redistribution is prevented by nicotinamide, a potent SIRT1 inhibitor [127].

#### 5.2.4. AKT

The serine threonine kinase AKT is a key component of the insulin signaling pathway whose downstream targets include regulators of metabolism, the stress response and apoptosis [128]. AKT is a negative regulator of PGC-1α. AKT positively regulates mTOR (activator of PGC-1α) by direct inhibition of the inhibitory complex TSC1/2 and by inhibition of the mTOR inhibitors AMPK (activator of PGC-1α) and GSK3β (regulator of PGC-1α turnover) [128,129].

#### 5.2.5. GSK3β

The nutrient sensitive kinase GSK3β (Glycogen Synthase Kinase 3 Beta) targets PGC-1α for nuclear proteasomal degradation [108], regulating PGC-1α turnover and thereby mitochondrial function and the process of aging. Regulation of PGC-1α turnover by GSK3β is also a component of the oxidative stress response in cultured cells and *in vivo*. GSK3β is downstream of the insulin and the Wnt signaling pathways both of which have been negatively associated with longevity [130,131].

Given the important role of circadian rhythms in various biological functions, cues from metabolic and nutrient status may influence biological clocks to modulate aging and disease processes. Studies in mammals have shown that rapamycin affects light entrainment of the mammalian central clock in the SCN in a phase dependent manner [132]. A study on *Drosophila* [133] also demonstrated that the TOR pathway can not only modulate entrainment of the clock but the free-running clock itself. This work supports the idea that nutrients may influence the central clock in flies and also add to the growing body of knowledge that clock genes play important roles in maintaining metabolic status and acting as an effector in modulating metabolic outputs.

## 6. Inter-Relationships between Metabolic Hormones, Circadian Timekeeping and Their Links to Aging

There are data available both from insects as well as vertebrate animal models that metabolic hormonal signaling pathways play a role in the circadian timekeeping mechanisms and in managing biological rhythms, which eventually may impact aging. Experimental evidences on the involvement of hormonal machinery in the control of circadian rhythms have been reported from several insects. It is supposed that a number of insect hormones—juvenile hormones (JHs), ecdysteroids and some neurohormones—control important biological rhythms in the insect body [134]. A bit surprisingly, the principal insect genetic model *D. melanogaster* does not seem to be the main focus, perhaps due to its small size for hormonal studies, and due to a weak effect of photoperiod on phenomena like diapause or reproduction, found in this species [135,136]. Recently, several publications appeared using the firebug *Pyrrhocoris apterus* (Heteroptera) addressing this topic. In *P. apterus*, adult diapause is primarily elicited by a short photoperiod and is controlled by JH, a product of the *corpora allata* endocrine gland [137]. It has been found in *P. apterus* not a long time ago that the expression of two circadian clock genes—*per* and *Pdp1* (par domain protein 1)—is controlled by photoperiodic signals [138]. An operation involving removal of active *corpora allata* from the experimental bugs significantly increased the expression of the *per* gene while the expression of the *Pdp1* was significantly suppressed by this intervention. These results were the first experimental proof for the effect of endocrine glands on circadian clock gene expression in insects.

Other candidates among the insect hormones that could play a role in the circadian rhythmicity are adipokinetic hormones (AKHs). These hormones are synthesized and released by the endocrine gland *corpora cardiaca* (CC) and represent a large neuropeptide family that is primarily responsible for mobilization of energy sources (lipids, carbohydrates, amino acids) in stress situations [139]. This role is accompanied by a plethora of functions on biochemical, physiological and behavioral levels (for review see [140]) including stimulation of heart beat, general locomotion and immune response. A rhythmicity of adipokinetic response was first demonstrated in the house cricket *Acheta domesticus* by Das and co-workers [141], who found that fat body sensitivity of this species to injection of Grybi-AKH varies in close synchrony with the lipid rhythm. In addition, the AKH content in the CC varies during the day with two peaks in the scotophase (*A. domesticus* is nocturnal), and one peak in the photophase. The locomotory activity of *A. domesticus* correlates with circadian changes in levels of neurosecretory proteins in neurosecretory cells of the brain [142].

A range of information demonstrating the role of AKH in a control of circadian rhythms was obtained in *P. apterus*. It is known that this species burns almost exclusively lipids and that injection of the Pyrap-AKH elicits lipid mobilization [143]. Maxová *et al.* [144] found that the maximal lipid mobilization in response to AKH occurs during photophase; this circadian rhythm correlates with locomotory activity that shows also the circadian rhythmicity whose endogenous nature has been demonstrated by Hodková [145]. In addition the rhythm in *P. apterus* locomotory activity is positively correlated also with the rhythmical changes of the AKH content in the CNS [146]. Also the AKH titer in the hemolymph of *P. apterus* fluctuates during a day [146], but no positive circadian correlation between this rhythm and fluctuation of the AKH content in CC has been recorded. It is supposed that the AKH level in hemolymph reflects the energetic needs of the body and is not directly under clock control. Nevertheless the clock control of AKH functions was demonstrated by the expression in the AKH-producing neurosecretory cells of the CC of the *per* gene [147], which is involved in the regulation of *P. apterus* circadian rhythms [148,149]. Additionally, the fact that the circadian changes in AKH level in CC, and in the mobilization of storage lipids and in the walking activity after the AKH treatment persist also in constant darkness [147] support the suggestion about the endogenous nature of AKH parameters.

Lee and Park [150] demonstrated that a free-running circadian rhythm of locomotory activity persists in *D. melanogaster* with deficiency of the AKH producing cells as well as in the wild-type flies. However, these authors recorded typical bimodal activity peaks in both groups that are sustained also in constant darkness, leading them to conclude that normal functions of AKH do not include the clock-controlled rhythms of locomotory activity in *D. melanogaster.* Recently, it was reported that overexpression of *dAKH* results in lifespan extension in *D. melanogaster*, and also that *dAKH* levels increase upon caloric restriction impacting lifespan [151]. It remains to be seen if *dAKH* is regulated directly by the clock or works independent of clock machinery directly impacting fat metabolism and thus aging. A similar effect of food restriction (FR) was observed in mice where FR resulted in elevated circulating levels of glucocorticoids correlated with enhanced lifespan [152]. Resetting of circadian timing in peripheral tissues by glucocorticoid signaling has been reported in mice models [153,154]. A robust phenotype associated with aging is a change in energy utilization, including body fat stores. Increases in body fat mass and deteriorations in insulin sensitivity are associated with aging in many mammalian species as well as in many clinical pathologies, including cardiovascular disease. Data obtained from clock gene mutants indicate a strong circadian mechanism regulating adipose stores, as well as the release of insulin and leptin, which activate anorexiegenic pathways of energy homeostasis and reduce food intake. The nocturnal rise in circulating leptin levels of younger animals are attenuated in older animals [155]. The amplitude as well as phases of metabolic hormones such as insulin, corticosterone and prolactin are disrupted in obese aged rodents, whereas administration of these hormones at specific times of day mimicking the rhythms of younger phenotypes re-establish the metabolic characteristics of younger animals [156]. Finally, there are very strong evidences of links between melatonin, clocks and aging [157]. These studies lead to the speculation of whether timed hormonal injections in aged populations would help in re-setting the energetic processes to allow for minimization of metabolic deficits. Such diverse approaches and studies in different model systems would be indicative of the complex interactions and inter-relationships between metabolic hormones, the clock machinery and the aging process.

## 7. Conclusions

The quest for new and effective means to delay aging or increase health span during the process of aging and thus delay the onset of aging associated diseases now has a new player in the arena—the circadian clocks. Mechanistic insights in the links between circadian clocks and aging process are being gleaned through studies in *Drosophila* as well as mammalian systems. With advancing age, animals exhibit numerous circadian disruptions [158–162] that contribute to poor health consequences and hastened senescence. While with age a marked dampening of the amplitude of oscillations of circadian clock transcripts is noticed [163], and longevity is diminished by mutations in clock genes [44], longevity can be decelerated by restoration of youthful circadian behavior by transplantation of a fetal clock into the brains of aged animals [164]. Thus, with increased understanding of the mechanisms driving age-related changes in clocks of various model organisms, novel insights concerning the nature of age-related pathologies resulting from loss of temporal precision can be gained. Aged individuals exhibit pronounced deficits in the amplitude and timing of core molecular clock genes and entrainment of the master clock to local time. This deterioration manifests as disruptions in sleep-wake cycle and system-wide physiology. Further studies on the links between circadian clocks and aging will afford greater insight into maximizing health with advancing age.
